# Tuberculosis meningitis coexisting with HIV Infection: a comprehensive review

**DOI:** 10.3389/ftubr.2023.1242869

**Published:** 2023-10-05

**Authors:** Inesa Navasardyan, Alexander Abdou, Samuel Kades, Yura Misakyan, Jacob Ochsner, Selvakumar Subbian, Vishwanath Venketaraman

**Affiliations:** ^1^College of Osteopathic Medicine of the Pacific, Western University of Health Sciences, Pomona, CA, United States; ^2^Public Health Research Institute, New Jersey Medical School, Rutgers, The State University of New Jersey, Newark, NJ, United States

**Keywords:** *Mycobacterium tuberculosis*, tuberculosis meningitis, HIV, opportunistic infections, immunosuppression, granuloma, antibiotics

## Abstract

*Mycobacterium tuberculosis* (*Mtb*) is the causative agent of tuberculosis (TB) in humans, Although *Mtb* is primarily considered a respiratory pathogen, its ability to spread to and affect the central nervous system (CNS) is of particular interest due to its clinical importance. Tuberculosis meningitis (TBM) is described as the manifestation of *Mtb* infection in the meninges, leading to inflammation and disease. Individuals with a weakened immune system, particularly those infected with human immunodeficiency virus (HIV), are more susceptible to both pulmonary and extrapulmonary *Mtb* infection. HIV infection leads to a gradual depletion of CD4 T-cells, severely impairing the host's immune response against pathogens and, thus, predisposes one to several opportunistic infections, including *Mtb*. Herein, we discuss the current knowledge, potential therapeutic agents, and mechanisms of action and describe various *in vivo* and *in vitro* models that may be used to study TBM coexisting with HIV infection.

## 1. Introduction

*Mycobacterium tuberculosis* (*Mtb*) is a bacterial pathogen responsible for causing tuberculosis (TB) in humans, which can present with detrimental pulmonary and extrapulmonary disease pathology (1). Approximately 4,000 deaths and 30,000 new cases are attributed to TB worldwide every day (2). *Mtb* is primarily transmitted via airborne particles, which travel through the epithelium of the lungs and infect host immune cells such as macrophages, dendritic cells, mast cells, and neutrophils (3). This process leads to the release of a plethora of cytokines and chemokines including tumor necrosis factor–alpha (TNF-α), interleukin 12 (IL-12), and IL-6, which serve as host defense mechanisms and leads to necrosis and apoptosis of *Mtb*-infected host cells. Eventually, the secretion of interferon-gamma (IFN-γ) and TNFα induce macrophage activation against *Mtb* causing both antibacterial and inflammatory events (4). The latter is responsible for granuloma formation, which serves as a barrier for infected cells and contains *Mtb* in a dormant and/or slow-replicating state in the initial infection foci, resulting in latent *Mtb* infection (LTBI) in the host. In immunocompromised patients, such as those with human immunodeficiency virus (HIV)/acquired immune deficiency syndrome (AIDS), the dormant pathogen may have the ability to transform into its actively replicating form and infect the lungs as well as other organs (5). TB is the primary cause of death among HIV-infected individuals, amounting to 214,000 in 2020 (2). The most common manifestation of TB is pulmonary tuberculosis (PTB), which is often initiated by *Mtb* establishing itself within lung alveoli, causing progressive disease. Persistent coughing accompanied by sputum production, chest pain, fatigue, weight loss, and night sweats are common signs of PTB (6). The pathogen invades macrophages, which may then spread to various areas such as the pleura, abdomen, gastrointestinal (GI) tract, bones, and meninges via the lymphatic system. The meningeal layers that are infected by *Mtb* can form Rich foci, which are ependymal cells that have the potential to burst out into the subarachnoid space and cause severe inflammation, resulting in the onset of meningitis. In severe cases, namely, in immunocompromised patients, *Mtb* infection may present with neurological symptoms caused by the increased influx of inflammatory cells (7). The diagnosis of *Mtb*-associated tuberculosis meningitis (TBM) involves the collection and culturing of cerebrospinal fluid (CSF) via a lumbar puncture. A positive result for *Mtb* infection is often accompanied by increased levels of proteins, white blood cells, and increased levels of adenosine deaminase (ADA) in the CSF; nevertheless, clinical presentation is frequently empirical to conclude the diagnosis of *Mtb*-induced meningitis (7). It is estimated that TBM constitutes ~2% of all TB cases among HIV-negative individuals and ~7% of those with HIV-TB. The mortality rates of TBM range between 20% and 70% despite standard anti-TB therapy, making TBM universally fatal if left untreated (8).

HIV is an enveloped double-stranded RNA retrovirus that is transmitted via unprotected sexual contact, contaminated blood transfusion, drug abuse, and from mother to child during birth. Through its complex mechanism, HIV attaches to CD4 T-cells and monocytes and utilizes co-receptors CCR5 and CXCR4 for host cell entry (9). The main surface glycoprotein 120 (gp120) present on the HIV envelope is responsible for invading the host immune cells, as well as releasing viral proteins and enzymes to initiate infection (10). As the virus obtains access into the cell, the viral reverse transcriptase releases ss-RNA to copy onto cDNA, which is predestined for erroneous transcriptions and mutations, making this virus increasingly resistant to treatment (11). When CD4+ T-cell counts have fallen below 200 cells/microliter, HIV infection is said to have progressed into AIDS, a condition characterized by a severe reduction in host defense mechanisms and increased susceptibility to opportunistic infections. It is worthwhile to mention that HIV can utilize microglial and other central nervous system (CNS) cells as its reservoir for survival and propagation. HIV has the potential to infiltrate through the blood-brain barrier (BBB) and disrupt its integrity, thereby establishing itself within the CNS. The complex array of nervous system-based issues stemming from HIV infection consists predominantly of conditions like HIV-associated neurocognitive disorders, opportunistic bacterial and fungal infections, and dementia that have been linked intrinsically with this chronic disease state (12). One of the outcomes associated with HIV infection is chronic immune activation accompanied by inflammation, in which severe depletion of CD4+ T-cells leads to worsening of disease progression. Current recommendations include the initiation of a four-drug anti-TB therapy, with isoniazid, rifampin, ethambutol, and pyrazinamide for TB, and the initiation of antiretroviral therapy (ART) in all patients with HIV-TB, regardless of their blood CD4+ cell count (13). Regardless of initiation of the anti-TB and ART treatment, patients have a poor prognosis and high mortality rate, particularly in TBM coexisting with HIV infection (14).

## 2. Current knowledge: coexistence of HIV and TBM

### 2.1. Relationship between HIV load and risk of TBM

The relationship between the viral load of HIV and the increased incidence of TBM is currently poorly understood. However, recent studies have illustrated several significant findings regarding the differences in plasma and CSF viral load in patients with and without TBM. One study analyzed ART-naïve patients diagnosed with TBM and found no significant differences in viral load between blood and CSF samples of patients with TBM (15). The study also did not reveal significant differences between TBM and non-TBM median plasma viral loads. Nevertheless, the study found that the median CSF viral load was notably higher in TBM patients compared to non-TBM CSF viral load (15). This suggests that the connection between TBM and heightened HIV replication in the CNS results from an elevated viral load, although a cause-and-effect relationship could not be established due to the cross-sectional design of the study. The literature has also suggested that patients with tuberculous or cryptococcal meningitis had the highest CSF viral loads in comparison to other forms of meningitis, including aseptic meningitis, tuberculoma, or AIDS dementia complex (16). The mechanism of interaction between TBM and the CNS continues to be a topic of further investigation, proposed to be a microbial synergy involving HIV–*Mtb* coinfection (17).

### 2.2. Relationship between levels of immunosuppression and prognosis of TBM

Macgregor and Vinnard suggest that a strong correlation exists between the level of immunosuppression and worsened prognosis of TBM in patients coinfected with HIV (14). HIV coinfection is associated with an increased risk for reactivation of LTBI as well as the rapid progression of primary infection into active TB. The literature describes a direct and positive correlation between an increased risk of active infection and a declining CD4+ count (14). Additionally, Macgregor and Vinnard illustrate the increased risk of extrapulmonary TB in patients who are coinfected with HIV (14). They also establish the correlation between the increased risk of extrapulmonary disease and TBM.

The increased incidence of active TB in individuals coinfected with HIV can be attributed to two primary mechanisms. Bruchfeld et al. (18) propose that, apart from elevating the risk of LTBI reactivation, the copresence of *Mtb* and HIV heightens vulnerability to opportunistic non-tuberculosis mycobacterial (NTM) infections. Thus, it is firmly established that HIV coinfection stands as the foremost risk factor for developing active TB, driven by the aforementioned mechanisms. A key facet of immunosuppression in AIDS patients involves a substantial reduction in CD4+ T-cells, directly contributing to the heightened active TB risk. Nevertheless, prior research indicates that the heightened susceptibility of HIV-infected individuals to active TB development occurs shortly after infection, preceding the CD4+ T-cell count drop below 500 cells/μL (19). This observation implies that other immune alterations and mechanisms play a role in the increased active TB susceptibility. Nonetheless, these factors remain inadequately understood and serve as subjects for future investigation (18).

**Figure 1:**
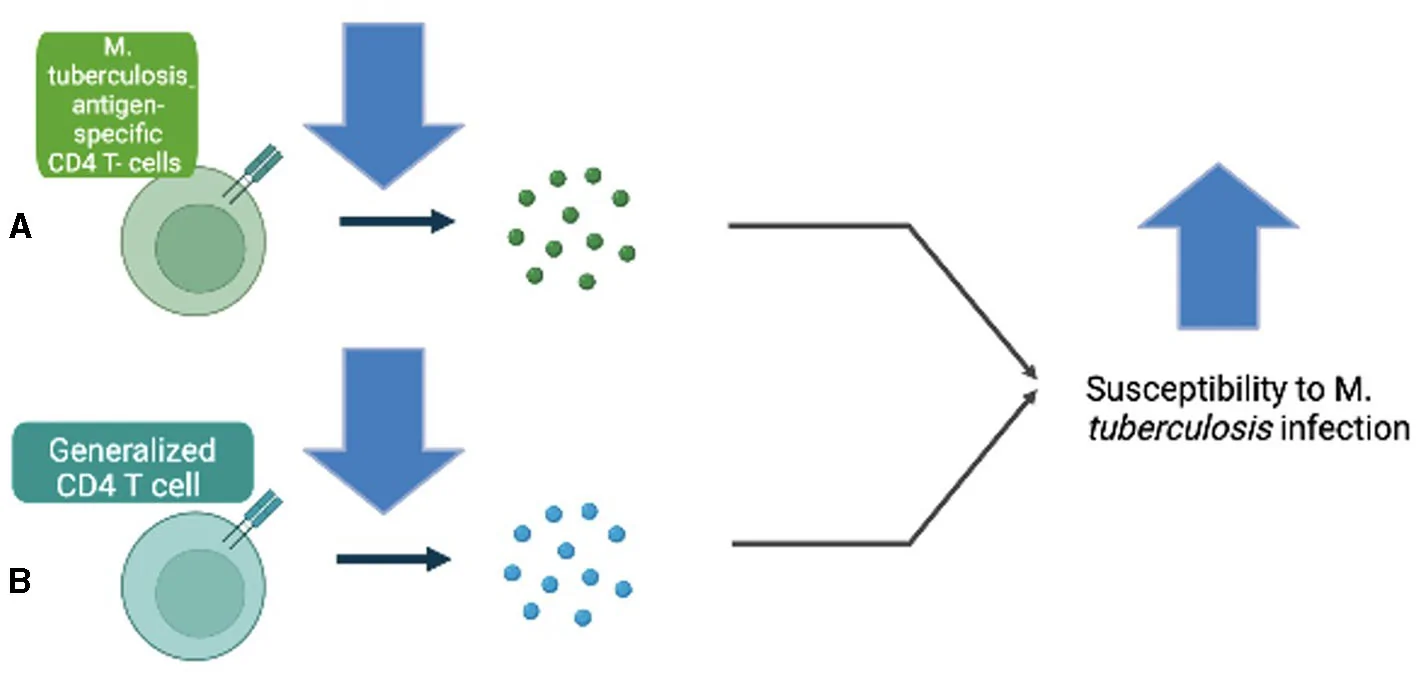
**(A)** Decreased *Mycobacterium tuberculosis* antigen–specific CD4 T-cells increase susceptibility to *M. tuberculosis* infection. **(B)** Decreased generalized CD4 T-cell count is also involved in increased susceptibility to *M. tuberculosis* infection.

A recent research hypothesis has proposed that HIV infection may alter *Mtb*-specific T-cells, which would contribute to increased susceptibility to active TB infection in patients who are infected with HIV (20). Geldmacher et al. demonstrated that a selective depletion of *Mtb* antigen–specific CD4+ T-cells occurs prior to generalized CD4+ T-cell depletion in patients infected with HIV (21) (Figure 1). Additionally, evidence suggests that a decrease in CD4+ T-cell count is correlated with increased susceptibility to developing HIV infection that produces IL-2, a critical component in a protective immune response against *Mtb* (18).

TNF-α, a general immune activation marker of the host inflammatory response, plays a dual role in susceptibility as well as resistance to TB infection. TNFα-mediated macrophage activation is crucial in the apoptotic response against *Mtb* infection; however, HIV infection impairs this response and, thus, renders HIV-infected individuals more susceptible to developing active TB (22). Moreover, TNF-α contributes to the formation and maintenance of granuloma cells in an effort to contain and limit the spread of *Mtb* infection. Anti-TNF-α therapy has been shown to prevent the maturation of granulomas and, thus, promote the free growth of *Mtb* (23). In HIV/TB coinfection, HIV-1 has been found to affect granuloma formation via disruption of TNFα, leading to increased dissemination of *Mtb* (24). Despite its protective effects during infection, high levels of TNF-α may lead to an overactive immune response, causing tissue damage and inflammation that contribute to disease progression (25). Infliximab, an inhibitor of TNF-α, has been successfully used to treat paradoxical TBM reactions in several cases, suggesting the involvement of TNF-α in disease pathology; however, the risks associated with infliximab use hinder its use in practice (26). Given its dual role in infectious disease, it is important to further explore the direct role of TNF-α in TBM.

The literature also suggests that *Mtb* infection negatively impacts the immune response to HIV by accelerating the progression of HIV infection to AIDS. Goletti et al. (27) demonstrate that the ongoing immune response against *Mtb* infection increases replication of HIV in the blood. Additionally, active TB is associated with the loss of CD4+ T-cells and an increased risk of opportunistic NTM infections (28). Immunosuppression is correlated with increased susceptibility to infection of *Mtb* and poorer prognosis. Therapeutic strategies, such as ART, suppress the replication of HIV by reducing viral load and increasing the number of CD4+ T-cells, thus reducing the risk for reactivation of LTBI into active TB, including TBM and the onset of NTM infections (18).

### 2.3. Current treatment strategies for TBM coexisting with HIV infection

Treatment strategies for HIV-associated TBM continue to be a significant topic of investigation within the literature. An empiric treatment plan for TBM without coinfection of HIV involves at least four first-line anti-TB drugs, classically involving 2 months of daily isoniazid, rifampin, pyrazinamide, and streptomycin or ethambutol followed by 7–10 months of rifampin and isoniazid (29). Additionally, complementary treatment with corticosteroids, such as dexamethasone, has been proven to improve mortality rates in patients with TBM due to the reduction of intracerebral inflammation (30). However, in TBM patients who are HIV positive, treatment approaches must consider drug–drug interactions, adverse immune reactions, and the role of intracerebral inflammation.

One effective treatment strategy includes reducing intracerebral inflammation and highly potent bactericidal agents that can penetrate the BBB. The current standard dose of rifampin has limited penetration in the CNS and rapidly declines in concentration shortly after initiation of treatment; thus, it has been suggested that an increased dose of rifampin could reduce the duration of TB treatment shortly after treatment initiation in animal models (31). Research studies yield conflicting results, however, as one study demonstrated intravenous (IV) administration of high-dose rifampin during the first 2 weeks of treatment substantially reduced mortality, whereas another randomized clinical trial did not illustrate a reduced mortality rate with oral rifampin treatment for TBM (32, 33). Furthermore, it has been suggested that outcomes in TBM may be more dependent on intracerebral inflammation than bactericidal activity (31). The literature suggests that increased dosages of anti-TB regimens may worsen intracerebral inflammation due to increased bactericidal activity, leading to an increased production of pro-inflammatory components; however, no significant difference in intracerebral inflammation associated with a high dose of rifampin was noted in the brain of mice infected with *Mtb* and treated with a high-dose rifampin regimen (31). Additionally, rifampin was detectable in the CSF of 71% of mice treated with high-dose rifampin and undetectable in mice with the standard-dose rifampin regimen, indicating the benefit of higher dosage in crossing the BBB and reducing bacterial burden (31). Another study on rifampin pharmacokinetic modeling and stimulation clearly indicates that the therapeutically effective dosing of rifampin would be achieved only with a high-dose treatment, suggesting that the standard dose of rifampin is suboptimal or ineffective in treating TBM cases (34). Researchers also studied the effects of dexamethasone, a corticosteroid included in the standard treatment of TBM, on a rifampin-containing regimen in mice. The results demonstrate that the bactericidal activity of rifampin was lower for dexamethasone-containing regimens, and in addition, lower brain rifampin concentrations were found in mice that received combination therapy with dexamethasone (31). However, decreased intracerebral inflammation, including IL-6 and IFN-γ levels, was noted in mice receiving dexamethasone plus rifampin, in contrast to those who were not treated with rifampin alone (31). Collectively, these findings demonstrate that high-dose regimens of rifampin may allow for efficient drug penetration through the BBB and reduced mortality in patients with TBM coexisting with HIV infection.

Chan and Marx demonstrate the risk of drug interactions and toxicity in concurrent ART and anti-TB therapy. The World Health Organization recommends that anti-TB therapy be started initially in a treatment regimen, followed by ART within 8 weeks (29). The sequenced order of treatment is due to rifampin, a critical component of TB treatment, inducing CYP-450 and thus metabolizing HIV ART, causing increased viral replication. Moreover, it is recommended that patients who have CD4 counts below 100 cells/μL begin ART after 2 weeks of anti-TB treatment (29). Further research is necessary to determine treatment regimens for multidrug-resistant forms of TBM.

## 3. Role of the BBB in TBM with HIV coinfection

The BBB lines the blood vessels of the brain and acts as a highly selective and semipermeable barrier to protect the brain. Substances such as water, oxygen, carbon dioxide, and general anesthetics are allowed entry, whereas toxins and foreign substances are often excluded from entering the brain. In the case of TBM, *Mtb* must disseminate from the respiratory epithelium and invade the BBB to establish infection in the meninges (35). One study highlights the ability of *Mtb* to cross the BBB in one of two mechanisms, either as free mycobacteria through systemic spread or through circulating *Mtb*-containing macrophages (35).

**Figure 2:**
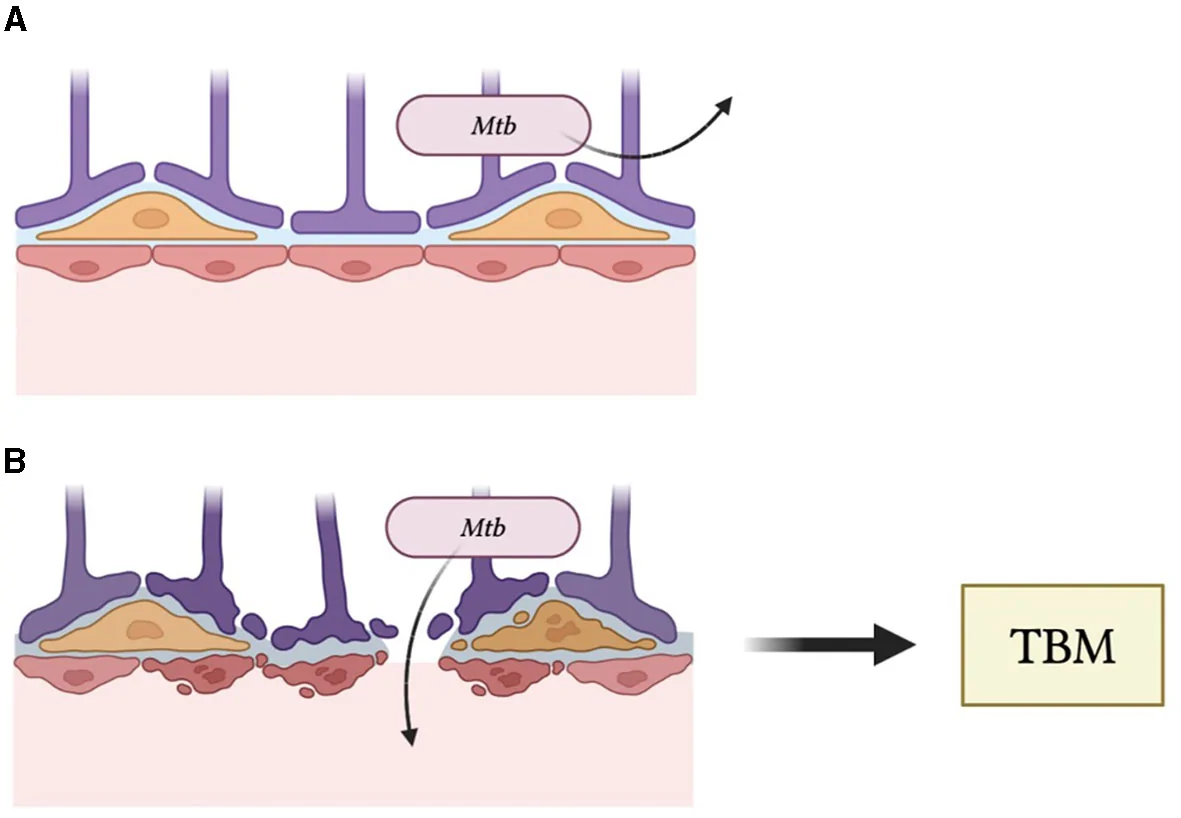
**(A)** The blood–brain barrier maintains the integrity of the central nervous system by excluding pathogens. **(B)** Human immunodeficiency virus infection causes disruptions of the BBB and, thus, reduces its ability to block entry of *Mycobacterium tuberculosis* (*Mtb*), leading to tuberculosis meningitis (TBM).

Studies have demonstrated that HIV-mediated disruption of tight junctions in the BBB results in its increased permeability to certain substances that would otherwise be excluded (36). Interestingly, the integrity of the BBB may be dependent on the duration of HIV infection and its associated viral load. Initially, the BBB acts as an effective barrier against viral particles; however, early infection with HIV may cause temporary dysregulation of the BBB until repair mechanisms act to reverse such damage (36). As infection ensues, the increase in viral replication and inflammatory cytokines associated with infection begin to irreversibly damage the BBB and reduce its effectiveness, allowing for invasion and infection of the CNS (36). In the case of HIV-positive individuals, HIV-mediated dysregulation of the BBB may be a potential mechanism by which *Mtb* can readily penetrate the CNS and manifest itself as meningitis (Figure 2).

## 4. Role of host defense mechanisms in TBM with HIV coinfection

Macrophages have been identified as the primary reservoir of HIV infection and have been proposed to transmit the virus to CD4+ T-cells, which are considered the primary target cells for HIV (37, 38). HIV infection is accompanied by a progressive loss of CD4+ T-cells, and it is this loss of CD4 T-cells that leads to its progression to AIDS. Reduced levels of CD4+ T-cells in the brain may account for the associated increased risk of TBM in patients infected with HIV. Notably, HIV patients with CD4+ T-cell counts in the normal range often exhibit pulmonary symptoms associated with TB infection. Conversely, as CD4+ T-cell counts fall below normal values, *Mtb* is more likely to disseminate and cause extrapulmonary infection (39). CD4+ T-cells and macrophages also serve as important host defense cells against *Mtb*, and thus, its reduced levels in HIV may account for the increased prevalence of TBM in these individuals. Resident macrophages of the brain, termed microglia, have been identified as the primary cell infected by HIV in the CNS and serve as a major cellular reservoir of latent HIV (40). Infection of microglia during HIV infection has been linked to the onset of neurocognitive symptoms observed in many HIV-positive patients. One study demonstrated upregulated microglia activation in a pediatric rabbit model of TBM, demonstrating the role of microglia as crucial neuro-regulators during CNS infection (41).

TNF-α is a proinflammatory cytokine that activates multiple signaling pathways to either induce activation or apoptosis of downstream target cells, mostly the innate phagocytes. Primary macrophages infected with HIV have been shown to secrete TNF-α (42). Moreover, TNF-α has been shown to contribute to HIV pathogenesis and expand the viral reservoir (43). An interesting relationship exists between the disease progression of TBM and TNF-α such that levels of TNF-α are positively correlated with the extent of pathogenesis of TBM (44). Further supporting this claim, the survival of experimental rabbits with TBM increased when treated with a combination therapy of antibiotics and thalidomide, a TNF-α inhibitor (45). Thalidomide has also been successful with humans in clinical trials; however, the teratogenic nature of the drug, as well as its ability to activate T-cells, limits its use (45). One could argue that the increase in TNF-α during HIV infection may also correlate with the pathogenesis and disease progression of TBM in HIV-positive patients. Furthermore, the development of a safer drug that similarly targets TNF-α may prove to be of therapeutic value.

Nitric oxide (NO) is a free radical that plays a crucial role in the host defense against pulmonary infections, including *Mtb*. NO may exert its bactericidal effects via its conversion into reactive nitrogen species. Increased levels of NO during infection occur via stimulation of inducible nitric oxide synthase (iNOS) by inflammatory stimuli. iNOS –/– mice infected with *Mtb* developed meningitis, highlighting the importance of NO in the CNS against *Mtb* infection (46). NO levels are significantly reduced in HIV patients (47). Moreover, levels of L-arginine, a precursor to NO, were found to be reduced in serum samples of HIV-positive patients (48). Given these findings, reduced levels of NO in patients with HIV infection may also account for the increase in *Mtb-*related meningitis in such patients.

## 5. Multidrug-resistant TBM in HIV-infected cases

Multidrug-resistant tuberculosis (MDR-TB) is classified by a shared ineffectiveness of at least rifampicin and isoniazid (49). Following treatment with these drugs residual organisms may modulate gene expression to limit repeat pharmaceutical efficacy. Studies have indicated that subsequent tolerance to rifampicin is, in part, attributed to a mistranslation of rpoB, in which upregulation contributes to metabolic shift. Similarly, upon exposure to isoniazid, changes in mycolic acid synthesis have been detected, circumnavigating the cell wall damage typically induced by isoniazid (50). These frontline drugs are preferred due to decreased toxicity and higher effectiveness in the treatment of Mycobacterium tuberculosis. Comorbidities such as HIV may pose an additional risk and complication for treatment, including increased mortality and progression of disease pathology (51).

Due to the recurrent and prolonged nature of MDR-TB in HIV-positive individuals, it is crucial to establish a thorough history of treatment to corroborate drug susceptibility testing (DST). A 2002 study in India demonstrated that certain strains of tuberculosis expressed *de novo* resistance, regardless of previous use, further highlighting the importance of appropriate DST (52). However, the paucibacillary nature of TB and LTBI can render rapid detection of drug resistance difficult (53). The current WHO recommendation for an initial diagnosis of MDR-TB coinfection with HIV is GeneXpert *Mtb*/RIF (54). This test allows rapid detection of Mycobacterium tuberculosis and identification of rifampicin resistance within 2 h with high specificity, albeit by using large volumes of CSF. In HIV patients, Xpert Ultra sensitivity was 70% compared to 43% for Xpert (55). High CSF bacillary load detected with these diagnostics has been associated with increased pathological severity and adverse neurological events within HIV-negative populations. Although this diagnostic is a powerful remedy to the inherent difficulty with diagnosis when positive, it is insufficient evidence to rule out tuberculous meningitis if negative. In this event, identification of Mycobacteria tuberculosis outside of the CNS, when clinically indicated, can assist with diagnosis (56). Another significant diagnostic, especially for extrapulmonary TB is adenosine deaminase (57). This test has been shown to be accurate for TBM diagnosis and is currently recommended by several representative medical associations (55). The necessity for prompt and accurate diagnosis, as well as subsequent appropriate treatment cannot be understated as it is a strong prognostic indicator regarding death and neurological deficit (55).

Attributable to the relatively rare cross section of the disease, the management of extrapulmonary MDR-TB is often extrapolated from data and clinical management of pulmonary MDR-TB (49). As such, the goal for treating MDR-TB is to limit the involvement of the meninges given that many of the preferred antibiotics have poor CSF penetration (53). Various factors, such as host defense mechanisms, may enhance susceptibility to meningeal TB, including a polymorphism in the toll-IL-1 receptor domain, and warrant additional research on immunomodulation as a mechanism of mitigating meningeal susceptibility (49). If indicated, localized pulmonary resection has been found to significantly improve outcomes (54).

A standardized treatment regimen for HIV MDR-TB is difficult to establish due to the relative drug susceptibility of the infection while also requiring a minimum of four effective drugs (58). The regimens are designed with a higher dose-intensive phase for a 5–7-month period followed by a 10–14-month prolonged maintenance phase. This timetable is established based on culture conversion (59). Current treatment regimens for HIV+ MDR-TB follow a longer protocol time, which may result from altered doses and drug–drug interactions with ART. Typically, treatment consists of a late-generation fluoroquinolone, bedaquiline, linezolid, cycloserine, and an injectable aminoglycoside, like amikacin or streptomycin, which demonstrates the ability to adequately treat the infection within the CNS (59). In contrast, other first-class drugs, namely, ethambutol provide little to no penetration and may lead to suboptimal concentrations in CSF once meningeal inflammation resolves (Mechai). This is clinically significant for treating MDR-TB, which has progressed to meningeal involvement (53).

Often, an all-oral treatment is preferred due to increased quality of life. If this can be accomplished without sacrificing efficacy or worsening toxicity, it poses itself as the potential treatment for future MDR-TB cases, even of a shorter duration (60). A shortened all-oral protocol, like those explored by endTB or the STREAM, would not operate on the clinical timetable as traditional treatment, although the application to TBM warrants further exploration (59). Other first-line drugs include ethambutol, and pyrazinamide, which are routinely less toxic and suitable bactericidal oral agents, for combination therapy. Capreomycin and fluoroquinolones when co-administered demonstrated an average increase in survival time of 20 weeks in a small study (61). In the Nix-TB trial, a 6-month administration of bedaquiline, pretomanid, and linezolid contributed to treatment success in 91% of HIV-positive individuals with non-responsive MDR-TB (57). The adoption of shorter, injectable-sparing protocols is likely to improve patient compliance and improve the logistical burden that can present, especially in lower socioeconomic areas, where this coinfection is most common (60). To briefly discuss mechanisms of action, pyrazinamide is a pro-drug and works well eliminating near-dormant bacilli within phagosomes or other acidic environments. While the direct mechanism is unclear, ethambutol inhibits the synthesis and formation of arabinan, a cell wall component. Understanding the interaction between cell wall synthesis inhibitors and latent residual bacilli provides a reasonable explanation for prolonged treatment.

Drug–drug interactions with ART warrant discussion regarding MDR-Tb and HIV-coinfected individuals. ART has been found to increase survival likelihood in MDR-TB patients affected by HIV and may be started within the first week of MDR-TB treatment (54), regardless of CD4+ count (62), a caveat being that the risk of early ART initiation is a complex immune reaction wherein a rapid expansion of TB-specific CD4 cells may precipitate an exaggerated innate immune response termed immune reconstitution inflammatory syndrome (57). This requires attentive clinical monitoring particularly with TBM, as it has been found to incur substantial morbidity and mortality (56). The protective effects of ART have been shown to reduce mortality rates in coinfected individuals over the duration of treatment; those who are severely immunosuppressed may take longer to respond to ART (62). MDR-TBM is considered the most lethal presentation of *Mtb*, and this is magnified within the HIV+ population. This reinforces the need for improved early detection and intervention in demographics most likely to demonstrate coinfection (51). Nevertheless, current data on HIV-positive MDR-TB reveals increased mortality, as well as poor prognosis when compared to HIV-negative counterparts, which may be attributable to a lack of adequate and available ART (62). Where ART is available, the standardized protocol consists of two possible regimens: zidovudine 300 mg/lamivudine 150 mg/nevirapine 200 mg given twice daily or tenofovir disoproxil 300 mg/lamivudine 300 mg/efavirenz 600 mg given once daily (63). Tenofovir has been found to interact with aminoglycosides, as such avoidance in simultaneous prescription is recommended. Efavirenz has been found to decrease serum bedaquiline concentrations, whereas protease inhibitors have been found to increase serum bedaquiline levels. Delamanid has shown no interactions or dose adjustments required for tenofovir or efavirenz (59).

It is critical to mention the relationships between areas where antiquated ART and those of MDR-TB treatment, due to restrictions in access. A comprehensive review of all patient's medications is warranted when beginning combination therapy to minimize the potential for toxicities (59). Second-line antibiotics demonstrate overlapping toxicities with ART and may include peripheral neuropathy, hepatotoxicity, and nephrotoxicity (61). For individuals newly exposed to ART, nucleoside reverse transcriptase inhibitors (NRTIs) may damage the mitochondria in outer hair cells, which may overlie ototoxicity due to complications through other drug–drug interactions (64). Didanosine, a component of ART, may include buffered tablets for proper absorption. However, the pH change limits the binding and gastrointestinal absorption of fluoroquinolones (61), which is regarded as the backbone of MDR-TB treatment. Moxifloxacin has demonstrated higher efficacy and may require less cardiac monitoring than other drugs in its class (65). A notable interaction between bedaquiniline and HIV protease inhibitors exists wherein the ART medication can increase bedaquiniline concentration and in turn, decrease the concentrations of Lopinavir. Due to this, if bedaquiniline remains a choice for first-line treatment a combination of two or three NRTIs with nevirapine may be done to maintain ART. Contrarily, Delamanid may be used as a first-line antibiotic and has shown no significant interactions with the CYP enzyme system or other ARTs (65). Clofazimine, a second-line treatment may inhibit CYP3A4/5 which is a critical site for maintaining adequate excretion of ART (65). The stigma, prolonged treatment, heavy pill burden, complex and delicate drug–drug interactions, immune system reconstitution, and multi-organ system ailments can lead to a resulting, yet understandable, poor compliance with the treatment plan. Unfortunately, this can result in totally drug-resistant tuberculosis, for which the outlook is even more grim.

Although the primary therapeutic regimen is complex and quite limited, especially for extrapulmonary MDR-TB there are adjunctive therapies that are clinically effective, albeit on a small scale. One of the primary causes of death in these patients is an ischemic stroke that may result from large-scale inflammation of the meninges. The time from diagnosis to death ranged from 1 week to 4 months (66). However, a randomized open-label placebo-controlled trial demonstrated that aspirin was efficient in reducing mortality to strokes within a 3-month period, resulting in a 36% decrease in mortality (61). The exact mechanism is not well understood but may be attributed to decreasing the inflammatory conductance of blood as well as decreasing the clotting factor cascade. An important sequela to meningeal inflammation can be poor CSF and cerebral venous drainage. This can result in seizures, poor temperature regulation, and respiratory difficulties (55). Imaging can assist with assessing edema and meningeal management, with computed tomography imaging providing the best opportunity to rule out emergent complications of TBM hydrocephalus (67). Reasonable evidence suggests that hydrocephalus can be managed with repeat lumbar punctures. If the condition instead obstructs the cerebral aqueduct or the outflow foramina, then an endoscopic third ventriculostomy may be indicated (55). Neurosurgical intervention has been shown to poorly improve prognosis (67). Steroids are a useful adjuvant for MDR-TB and should be administered in extrapulmonary patients as they decrease overall inflammation and potentiate the limited CSF-penetrating antibiotics when clinically indicated (49). Adjunctive dexamethasone has been shown to significantly reduce mortality (53). In select cases, TNF-α inhibitors have been shown to decrease the paradoxical reaction. Infliximab, a powerful tumor necrosis factor was given to a patient with hemorrhagic meningitis and was found to significantly reduce inflammation visible to imaging (49). A precautionary Bacillus Calmette–Guérin (BCG) vaccine has shown a strain-sensitive protective effect for meningeal involvement of MDR-TB; however, this may be less effective in immunosuppressed populations (53).

Another significant component of a sustainable treatment protocol is setting. One protocol established in Lesotho, South Africa, consisted of a specialized and centralized patient program developed to monitor the progress of individuals coinfected with HIV and MDR-TB. Through direct observational therapy, clinical staff recognized and reported signs and symptoms of adverse treatment or pathological effects (62). Although resource-intensive, it was shown to be highly effective and reported one of the lowest mortality rates in coinfected individuals. In contrast, another protocol from rural South Africa consisted of treatments performed strictly at home. In this study, a dedicated team of community health workers and nurses were trained to administer ART and comprehensive MDR-TB treatment in individuals' homes in hopes of eliminating mechanical and financial barriers to receiving treatment. Following treatment, a brief questionnaire regarding symptoms and side effects was administered, and more severe cases were brought to the clinic for immediate evaluation (68). This proved to be a promising model for delivering care to remote areas with a high prevalence of disease and boasted an impressively low mortality rate. Both models aimed to combat adherence, a crucial and difficult factor to manage in successful treatment. While treatment strategies remain promising, there exists a need for further research in identifying therapeutic agents in individuals with established extrapulmonary MDR-TB.

## 6. Potential models to study TBM with HIV coinfection

Murine models appear to be a promising tool in studying the pathogenesis of TBM in HIV-coinfected patients. *In vivo* data demonstrates that mice can be intracerebrally injected with *Mtb* and that the bacilli could be found in brain homogenates replicating 24 weeks post-infection (69). Upon measuring the chemokines and cytokines in murine serum and brain homogenates, they were unable to find elevated cytokine levels typically associated with TBM but found increased chemokines that have leukocyte-recruiting affinity (69). The researchers attributed the absence of change in cytokine levels to the killing of the mice 3 weeks post-infection due to disease complications. It was reasoned that the cytokine levels mimic human TBM infections, which have shown an increase in cytokine levels for up to 7 days (70). While this model is reproducible, it is not capable of replicating the natural mechanism or symptoms of *Mtb*–HIV coinfection in humans and has very different cytokine expression profiles compared to humans (69). To better understand the immunopathogenesis of TBM in HIV-coinfected patients, this model can be combined with existing humanized mice models or non-human primate models that effectively analyze the immunopathogenesis of HIV.

Zhang and Su first studied HIV immunopathogenesis in severe combined immunodeficiency (SCID) humanized mice by injecting human peripheral blood mononuclear cells (PBMCs), a mixture of different stem cells typically found in the marrow as well as mature and immature cell types of myeloid, lymphoid, and erythroid lineages (71). They successfully established an HIV infection that resulted in CD4+ T-cell depletion and non-specific immune response activation, which mimics the observation in human patients. To study *in vivo* HIV infections, Fan et al. have established a new humanized murine mouse model in severe immune-defect phenotype B-NSG mice (NOD-PrkdcscidIl2rgtm1/Bcge) (72). These mice have shown longer life spans than classical SCID mice used in the last 3 decades, making them more ideal for human cell engraftment (73). The B-NSG mice engrafted with human PBMCs via mouse tail vein injection were repopulated 3 weeks post-transplantation with human CD45+ (hCD45+) leukocytes. The hCD45+ cells were mainly composed of CD4+ and CD8+ T-lymphocyte subsets in the peripheral blood of mice (72). The newly formed CD4+ T-cells expressed the appropriate HIV co-receptor CCR5, but not CXCR4, which made it susceptible to CCR5-tropic HIV-1/JR-CSF–induced viremia. HIV infection resulted in the expected T-lymphocyte dynamics observed in human patients and subsequent restoration of levels with antiretroviral therapies, rendering the model successful (72). However, the humanized mice model remains impractical such that the human PBMCs engrafted can only differentiate into human T-cells and not the cells that support T-cell function. The potential to modify the strategy by co-injecting the PBMCs with human CD34+ hematopoietic stem cells may provide a solution to this limitation and improve the reconstruction of elements of the human immune system both in tissues and peripheral blood (74, 75). The rapid and convenient humanized B-NSG mice model can be used to study T-cell dynamic changes and novel treatment modalities in patients and therefore may be a promising tool to also study TBM with HIV coinfection.

Non-human primate models are also being used to help discover a cure for HIV. Indian rhesus macaques (RM) or pigtail macaques (PTM) infected with simian immunodeficiency virus (SIV) or simian/human immunodeficiency virus (SHIV) when treated with ART have shown to have a similar pathology and disease progression when compared to ART-treated HIV infections in humans (76–78). In addition to studying the effectiveness of ART, the model is useful for studying viral transmission, pathogenesis, antibodies, and candidate vaccines (76–78). However, limitations do exist with these models such that researchers are unable to examine human-specific immune functions due to the genetic differences in the major histocompatibility complex genes of the non-human primates. It is also difficult to gather sufficient sample sizes due to their expensive cost and the ethical concerns related to the experimentation with large animals (79). Due to these concerns, the use of B-NSG humanized mice models seems more promising for studying TBM/HIV coinfections.

The discovery of an *in vitro* model that can represent HIV CNS infection in humans has been challenging yet plausible with the incorporation of revolutionary organoid systems. One of the first HIV-infected organoid studies performed by Dos Reis et al. (80) successfully incorporated HIV-infected microglial cells into a 3D human brain organoid model and were able to observe the respective changes in the inflammatory response molecules, such as TNF-α and IL-1β post-infection. This study, however, required the infection of microglia prior to its incorporation in the organoid, which made it harder to draw comparisons to an *in vivo* model in which the HIV-susceptible microglial cells would be surrounded with other CNS cell types pre-infection (80).

Gumbs et al. (81) reported an HIV infection model within microglia-containing cerebral organoids encompassing all the major CNS cell types. They demonstrated the ability to reproduce a productive HIV infection in organoid-derived microglia (oMG), two-dimensional (2D) organoid dissociates, and three-dimensional (3D) organoids using the replication-competent HIV reporter viruses. The researchers also showed that the microglial viral infection was mediated by the CD4 and CCR5 co-receptors (81).

While utilizing an organoid model seems promising, there are some drawbacks in terms of available resources and data collection. In comparison to cultured human primary microglia that have expressed continuous viral spread and production, the oMG, 2D organoid dissociates, and 3D organoids have much lower infection rates (81). In addition to the low number of infected cells, a well-known difficulty when analyzing HIV in the cerebral organoid model is the limited number of microglial cells and their various stages of development during HIV infection (81). Despite these challenges, the organoid model that is being explored can be useful to further study HIV infections in the CNS, better understand its neuropathogenesis, and test new therapeutic interventions.

## 7. Conclusion

In this review, we summarized the impact of TBM, which occurs at a greater incidence in HIV-coinfected patients due to their immunosuppressed states. We further discussed the pathological mechanisms underpinning TBM with HIV coinfection in humans and the gold standard treatment regimens used in clinical practice. We have highlighted the potential implications of multidrug-resistant TBM as well as the adaptive therapeutic regimens currently in use. We have also explored the future modalities being investigated through *in vivo* and *in vitro* models researchers have developed to study both disease processes within the last decade.

Although the primary therapeutic regimen for TBM with HIV coinfection is complex and quite limited, adjunctive therapies may prove to be clinically effective, albeit on a small scale. One of the primary causes of death in these patients was ischemic stroke, which may result from large-scale inflammation of the meninges. The time between diagnosis and death ranged from 1 week to 4 months (66). However, a randomized open-label and placebo-controlled trial demonstrated that aspirin was efficient in reducing the mortality of strokes within 3 months, resulting in a 36% decrease in mortality (61). The exact mechanism is not well-understood but may be attributed to decreasing the inflammatory conductance of blood, as well as decreasing the clotting factor cascade. Similarly, steroids are a useful adjuvant for MDR-TB and should be administered in select extrapulmonary TB cases as they decrease overall inflammation and potentiate the limited CSF-penetrating antibiotics (49). Another potential adjunctive agent is glutathione, an endogenous compound that can reduce oxidative stress. It has been shown to reduce granuloma formation and subsequent liquefaction within immunocompromised individuals, a process that is mediated by TNF-α, IL-2, and various other host immune components (82). Glutathione, when potentiated with IL-2 and IL-12, was noted to increase the activity of natural killer cells and inhibit *Mtb* growth (82).

Nevertheless, there still exists a need for further research to better understand the underlying mechanisms and develop potential therapeutic strategies against TBM coexisting with HIV. For instance, given the role of oxidative stress in the progression of TBM in healthy and HIV-coinfected subjects, we propose the use of glutathione in adjunct treatments. Such a treatment is hypothesized to enhance both the innate and adaptive immune response of the host immune system, resulting in an improved prognosis and prevention of *Mtb* and/or HIV infection. Additionally, we also propose the combination of various murine models in effect to study the *Mtb*-HIV coinfection *in vivo*. A promising next step could involve combining aspects of both the intracerebral *Mtb* infection model as well as the B-NSG humanized mice model to further refine the HIV/*Mtb* coinfections, which may provide greater insight into the understanding of the pathogenesis of TBM in HIV-coinfected patients. We also anticipate that organoids to be a revolutionary *in vitro* model, effective in emulating the CNS environment needed to accurately simulate the localized coinfection in humans.

## Author contributions

Conceptualization: VV and IN. Software: IN. Writing: IN, AA, SK, YM, JO, and SS. Supervision: VV. All authors have read and agreed to the published version of the manuscript.
